# Human RNASET2 derivatives as potential anti-angiogenic agents: actin binding sequence identification and characterization

**DOI:** 10.18632/oncoscience.100

**Published:** 2014-11-26

**Authors:** Liron Nesiel-Nuttman, Shani Doron, Betty Schwartz, Oded Shoseyov

**Affiliations:** ^1^ The Robert H. Smith Institute of Plant Science and Genetics in Agriculture, The Robert H. Smith Faculty of Agriculture, Food and Environment, The Hebrew University of Jerusalem, Rehovot, ISRAEL; ^2^ School of Nutritional Sciences Institute of Biochemistry, Food Science and Nutrition, The Robert H. Smith Faculty of Agriculture, Food and Environment, The Hebrew University of Jerusalem, Rehovot, ISRAEL

**Keywords:** actin-binding, antiangiogenesis, ribonuclease, peptides, RNASET2

## Abstract

Human RNASET2 (hRNASET2) has been demonstrated to exert antiangiogenic and antitumorigenic effects independent of its ribonuclease capacity. We suggested that RNASET2 exerts its antiangiogenic and antitumorigenic activities via binding to actin and consequently inhibits cell motility. We focused herein on the identification of the actin binding site of hRNASET2 using defined sequences encountered within the whole hRNASET2 protein. For that purpose we designed 29 different hRNASET2-derived peptides. The 29 peptides were examined for their ability to bind immobilized actin. Two selected peptides-A103-Q159 consisting of 57 amino acids and peptide K108-K133 consisting of 26 amino acids were demonstrated to have the highest actin binding ability and concomitantly the most potent anti-angiogenic activity.

Further analyses on the putative mechanisms associated with angiogenesis inhibition exerted by peptide K108-K133 involved its location during treatment within the HUVE cells. Peptide K108-K133 readily penetrates the cell membrane within 10 min of incubation. In addition, supplementation with angiogenin delays the entrance of peptide K108-K133 to the cell suggesting competition on the same cell internalization route. The peptide was demonstrated to co-localize with angiogenin, suggesting that both molecules bind analogous cellular epitopes, similar to our previously reported data for ACTIBIND and trT2-50.

## INTRODUCTION

The existence and progression of the cancerous processes requires new growth in the vascular network since adequate proliferation, as well as extensive metastatic spread of cancer cells, depends on sufficient supply of oxygen and nutrients and the removal of waste products. The formation of new blood vessels from pre-existing dormant vasculature is termed angiogenesis [1]. The progression of the carcinogenic process depends on both the motility of the cancer cells forming metastases and the motility of the endothelial cells creating new blood vessels. The motility of cells requires the formation of actin-rich cell protrusions called phyllopodia and lamellipodia [2,3]. Therefore, these actin-rich pseudopods are a prerequisite for cancer-cell function. Endothelial cell proliferation, migration, and actin reorganization, are all necessary components of an angiogenic response [4]. Pathological angiogenesis is necessary for tumors and their metastases to grow beyond a microscopic size and it can give rise to bleeding, vascular leakage and tissue destruction [5]. To ensure an adequate blood supply, tumor cells release angiogenic factors that are capable of promoting nearby blood vessels to extend vascular branches towards the tumor. Angiogenesis is a complex multistep biochemical process, and offers several potential molecular targets for non-cytotoxic anticancer therapies [6]. In comparison to tumor cells, endothelial cells are considered to be relatively genetically stable [7,8], therefore direct angiogenesis inhibitors could prevent acquired drug resistance [9]. The human RNASET2 (hRNASET2) protein has been previously shown to inhibit angiogenesis and consequently tumorigenesis via a mechanism associated with specific binding to cellular actin [10]. Ribonucleases (RNases) are very important enzymes for RNA metabolism found in almost all organisms [11]. Studies on RNases began with the discovery of a thermostable enzyme in the extracts of bovine pancreas that hydrolyzed RNA [12]. Traditionally, RNases are classified by different ways according to base specificity, structure, function, optimal pH, and origin [13]. In broad terms, RNases can be classified as alkaline RNases (including the RNase T1 and the RNase A families) and acid RNases comprising essentially the RNase T2 family [11]. Comparison of the crystal structures of T2-RNases revealed that the overall structured of the RNases are similar except for some differences in the exposed loop regions. Their core includes the conserved active site that is responsible for degrading RNA [14,15].

Human RNASET2 is a glycoprotein encoded by the *RNASET2* gene which is located on chromosome 6 (6q27) and demonstrated to be a tumor suppressor gene [16-20]. RNASET2 is the only member of the Rh/T2/S family of acidic hydrolases in humans [21] and shares 34% identity and 52% similarity with ACTIBIND (a fungal RNase T2).

In addition to their ability to control RNA metabolism, certain RNases display a variety of biological activities. In tumor cells, RNases uniquely influence several functions simultaneously. They demonstrate the ability to overcome multi-drug resistance and to enhance the cytotoxicity of a variety of anti-cancer agents. The RNase-based mechanism thought to drive their cytotoxic effect in their ability to adsorb specifically to certain cells, enter their cytosol, degrade the RNA and thereby inhibit protein synthesis, and ultimately cause cell death [22]. Therefore, RNases' therapeutic potential is limited primarily by their ability to penetrate the cell.

The mechanisms of action attributed to both ACTIBIND and hRNASET2 are quite different. Their effect is mediated primarily through binding to cellular actin present at the cell surface leading to interference with organization of the actin cytoskeleton, which finally affects cell migration. This function seems to be independent from their RNase activity [3,10,22-25]. One of the aims of the present study therefore is to establish the actin binding sequence of hRNASET2. Determination of the actin binding sequence enabled us to develop shorter, but still biologically active peptides. These actin-binding peptides were demonstrated to inhibit angiogenesis, similarly to the hRNASET2 protein.

Gundampati et al. 2012, and Kumar et al. 2013 [26,27] examined the mechanism associated with binding of ACTIBIND to human actin using *in vitro* and *in silico* studies. The docking of ACTIBIND and actin revealed that the amino acids T L D S Y T A L S D A G I T P S E D A T Y K play a role in actin binding. Several amino acids residues, including: T L D Y T S D I T P E D, were identified to exclusively contribute to the binding. The efficient binding of *A. niger* RNase and human actin suggested that the amino acids S E D A T Y K are even more specific to bind actin.

**Figure 1:**
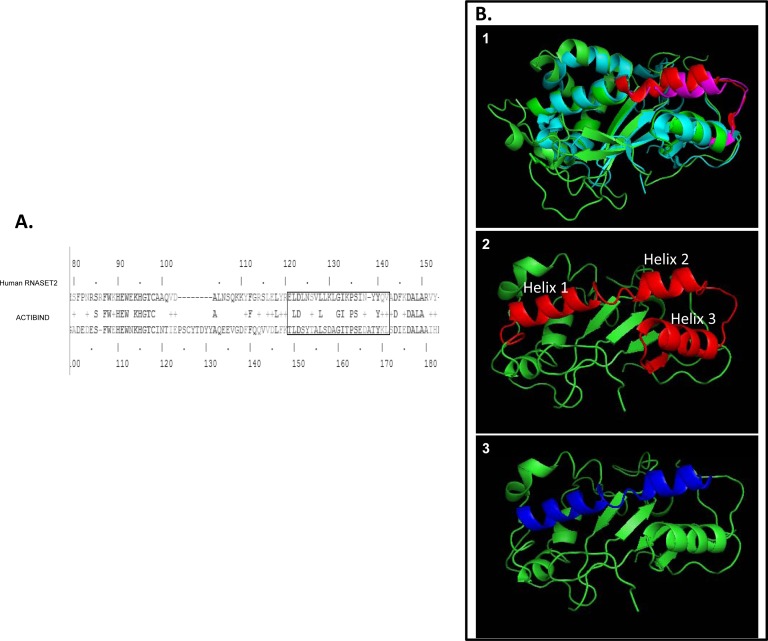
**(A): Sequence alignment of ACTIBIND and human RNASET2.** The box represents the sequence identified by Gundampati et al. 2012 to bind actin and the homologous sequence on the human protein sequence [26]. According to this comparison, residues 120-141, and specifically residues 135-141, were identified to exclusively contribute to the binding of hRNASET2 to actin. **(B): Structural analysis using PyMole (an open-source and widely used biomolecular visualization program) and based on Thorn et al. 2012** [21]. 1: ACTIBIND and the hRNASET2 structures. In green-ACTIBIND. In light blue-hRNASET2. In pink-ACTIBIND sequence identified by Gundampati et al. 2012 to bind actin (T149-L171) [26]. In red-hRNASET2 homologous sequence (E120-A141). 2: hRNASET2 structure. In red- the longest peptide consists of 57amino acids (A103-Q157) that was identified in this work to bind actin and to inhibit angiogenesis. 3: hRNASET2 structure. In blue- the shortest peptide consisting of 26 amino acids (K108-K133) that was identified in this work to bind actin and to inhibit angiogenesis.

Based on the homologous sequence of the human RNASET2 (Fig. 1A) and based on structural analysis (Fig. 1B), we have generated a peptide library consisting of 29 peptides. In the structural analysis of ACTIBIND and hRNASET2 it is shown that both proteins have high structural similarity in the area of A103-Q159 amino acids (hRNASET2 numbering, Fig. 1B). In fact, this was the first peptide that was synthesized and found to bind actin. Helical wheel prediction revealed that out of the three helixes within this peptide, the first helix has three positively charged amino acids facing out, whereas for the other two only one positively charged amino acid is facing out. This has led us to generate a peptide library and establish the shortest peptide able to bind actin and concomitantly inhibit angiogenesis (Supplementary table S1).

## RESULTS

### Affinity chromatography purification of trT2-50m

**Figure 2:**
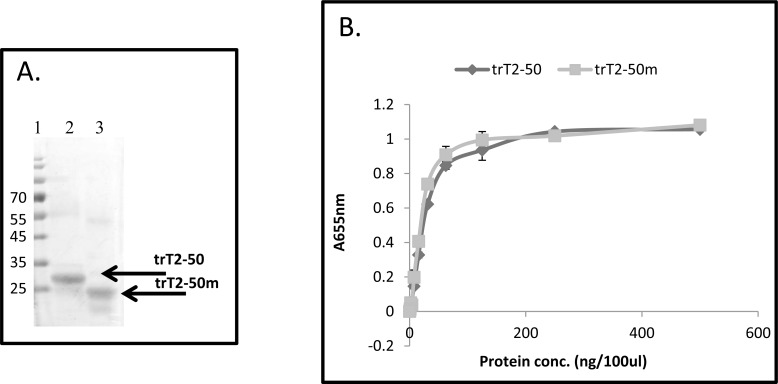
**(A): Purified trT2-50 and trT2-50m on a 12.5% SDS-PAGE.** Lane 1: molecular markers. Lanes 2: purified trT2-50. Lane 3: purified trT2-50m. **(B): trT2-50m binds actin on solid phase**. trT2-50 or trT2-50m were added to immobilized actin at increasing concentrations from 0 to 500 ng/100μl/well, and then reacted with rabbit anti-trT2-50 and goat anti rabbit IgG-HRP. The amount of bound protein was quantified by measuring the optical density at 655nm. Optical density was elevated in correlation to trT2-50 and trT2-50m increasing concentrations.

The truncated version of hRNASET2 missing the sequence of ELDLNSVLLKLGIKPSINYYQV amino acids, termed trT2-50m, was optimized for *E. coli*. Recombinant trT2-50m, expressed in *E. coli*, was purified on a HisTrap affinity column and analyzed by SDS-PAGE. A pure protein at the expected size of about 25 kDa (Fig. 2A) was eluted in about 75 mM imidazol. Following protein purification and refolding, the protein was lyophilized and the yield was ~ 15 mg/100 ml growth medium.

### trT2-50m binds actin *In vitro*

Binding of trT2-50m to actin was analyzed by solid-phase actin binding assay. trT2-50m bound actin in a concentration-dependent manner (Fig. 2B) with a binding affinity of 34.5 × 10^−9^ M. This actin binding capability is similar to that of trT2-50 [25]. To further evaluate the strength of this interaction we performed BIAcore analysis in order to quantify and understand the kinetics of the interaction between these two molecules. Using actin coupled to a biosensor chip, the affinities of trT2-50 and trT2-50m were measured. Similar affinity constants were measured for both proteins. A summary of the derived constants is shown in Table 1. Furthermore, there is a linear relation between the protein concentration and the maximal (steady-state) response for hRANSET2, trT2-50 and trT2-50m, indicating the pseudo-first-order regime in relation to the immobilized actin. Altogether, these results indicate that the ELDLNSVLLKLGIKPSINYYQV protein sequence is not essential for actin binding.

**Table 1:** Affinities constants measured by BIAcore analysis. Summary table of the affinities measured by BIAcore analysis

	Ka × 10^3^ (1/Ms)	Kd (1/s)	KD × 10^−6^ (M)
hRNASET2	2.40	0.00346	1.44
trT2-50	7.72	0.0132	1.71
trT2-50m	19.4	0.00969	0.5
Peptide A103-Q159	3.04	0.00322	1.06
Peptide K108-K133	14.2	0.0458	3.32

Actin from rabbit muscle (Sigma A2522) was coupled to a CM5 biosensor chip (GE Healthcare Bio-Sciences AB). The binding assay was performed by injecting the hRNASET2 (Produced in CHO, T2BIOTECH Ltd), trT2-50, trT2-50m (Produced in *E.coli*, self-production) or peptides (Genemed Synthesis Inc.; Tx USA, by HyLabs, Israel) at 5 different concentrations at a flow rate of 10 μl/min at 25°C. The association (Ka) and dissociation (Kd) rates and dissociation constant (KD), were determined from kinetic analyses performed as outlined in the methods section. All binding data were analyzed using BIAevaluation 3.2 software.

### Peptides A103-Q159 (57aa) and K108-K133 (26aa) bind actin *in vitro*

**Figure 3:**
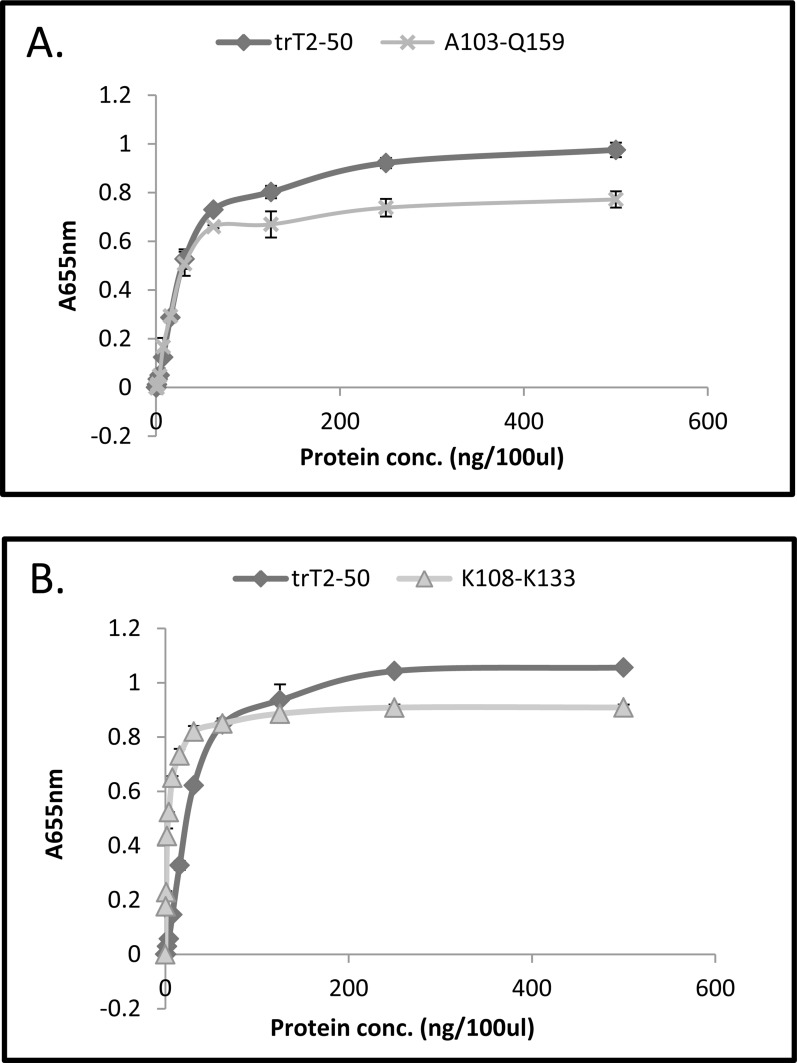
Peptide A103-Q159 (57aa) (A) and Peptide K108-K133 (26aa) (B) binds actin on solid phase trT2-50 or the peptides were added to immobilized actin at increasing concentrations from 0 to 500 ng/100μl/well, and then reacted with rabbit anti-trT2-50 and goat anti rabbit IgG-HRP. The amount of bound protein and peptides was quantified by measuring the optical density at 655nm. Optical density was elevated in correlation to trT2-50 and peptides increasing concentrations.

For further insight on the actin binding site within the hRNASET2 protein sequence we tested 29 suspected peptides for their ability to bind actin. The actin-binding capability of the 29 synthetic peptides was compared to the binding capability of trT2-50 using a solid phase actin binding assay. The results obtained demonstrate that the peptide A103-Q159 and the peptide K108-K133 are the most effective actin binding peptides amongst the 29 tested peptides (Fig. 3) with affinities of 68 × 10^−9^ M and 10.5 × 10^−9^ M, respectively. We next tested the interaction kinetics to evaluate the interaction nature as described for trT2-50 and trT2-50m. Similar affinity constants (same order) were measured for peptides A103-Q159 and K108-K133 (Table 1). In addition, there is a linear relation between the protein concentration and the maximal (steady-state) response for peptides A103-Q159 and K108-K133, indicating the pseudo-first-order regime in relation to the immobilized actin.

### trT2-50m and Peptides A103-Q159 (57aa) and K108-K133 (26aa) inhibit HUVEC tube formation on Matrigel

**Figure 4:**
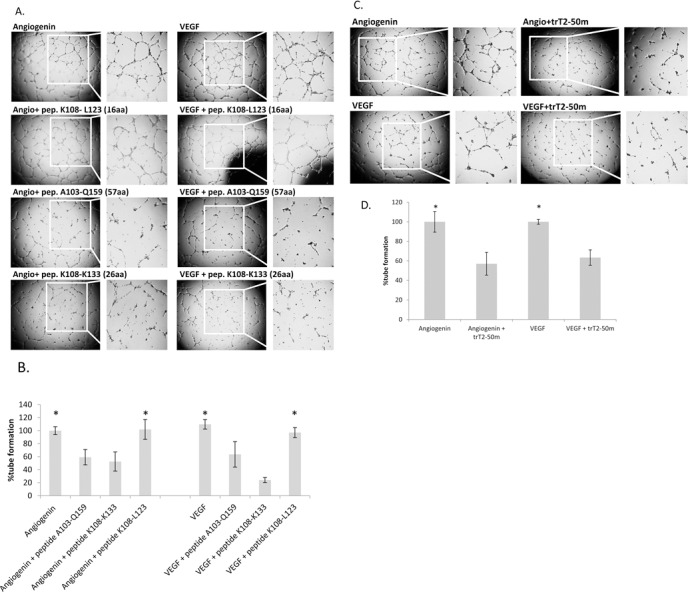
trT2-50m and peptides A103-Q159 (57aa) and K108-K133 (26aa) inhibit angiogenin- and VEGF-induced HUVEC tube formation on Matrigel Freshly isolated HUVECs were plated in a 96-well plate previously coated with Matrigel. (A): Cells were treated with either peptide A103-Q159, K108-K133 or K108-L123 (2μM) or PBS (control), in addition to angiogenin or VEGF (1 μg/ml). (B): tube formation was assessed using *Image J*, the results are represented as percent of control. Peptides A103-Q159 and K108-K133 had a significant inhibitory effect on tube formation in the presence of angiogenin and VEGF. (C): Cells were treated with trT2-50m (2μM) or PBS (control), in addition to angiogenin or VEGF (1 μg/ml). (D): tube formation was assessed using *Image J*, the results are represented as percent of control. trT2-50m had a significant inhibitory effect on tube formation in the presence of angiogenin and VEGF. *Represents a statistically significant difference between − and +, as analyzed using one way ANOVA for multiple comparison and 2-sample t-test, *P < 0.05*.

The antiangiogenic effect of trT2-50m and peptides A103-Q159 and K108-K133 was assessed in a HUVEC tube formation assay. The antiangiogenic effect was compared to the effect of peptide K108-L123 (16 aa) that failed to bind actin and to a control (PBS). Treatment with 2μM peptide A103-Q159 led to statistically significant ~40% inhibition (*P* < 0.05) of angiogenin- and VEGF – induced tube formation compared to the Control (Fig. 4A and B). Treatment with peptide K108-K133 led to statistically significant ~50% inhibition (*P* < 0.05) of angiogenin –induced tube formation (Fig. 4A and B) and ~75% inhibition of VEGF –induced tube formation compared to the Control (Fig. 4A and B). Peptide K108-L123, in addition to failing to bind actin, was also unable to inhibit tube formation (Fig. 4A and B). Treatment with trT2-50m led to statistically significant 40% inhibition (*P* < 0.05) of angiogenin –induced tube formation (Fig. 4C and D) and to statistically significant 35% inhibition (*P* < 0.05) of VEGF –induced tube formation (Fig. 4C and D), (N=5 for each treatment).

### Peptide A103-Q159 (57aa) and peptide K108-K133 (26aa) inhibit blood vessels formation in an *Ex Ovo* CAM assay, similar to the hRNASET2 and trT2-50

In this assay we used fertilized eggs cracked into a petri dish after 3 days of incubation in 37°C and 60% humidity. In the following days, the embryos developed a vascular network. Treatment began on the eighth day of incubation and was delivered drop-wise onto disks of Whatman paper that had been placed on the developing vascular regions. The treatment was performed every day for 4 days. The number of blood vessels around the treated disk was counted every day. In the disks treated with either one of the two peptides, the hRNASET2 or trT2-50, fewer blood vessels developed compared to the control (Supplementary Fig. S1).

### Immunofluorescence

#### Peptide K108-K133 (26aa) rapidly internalizes into the cell

**Figure 5:**
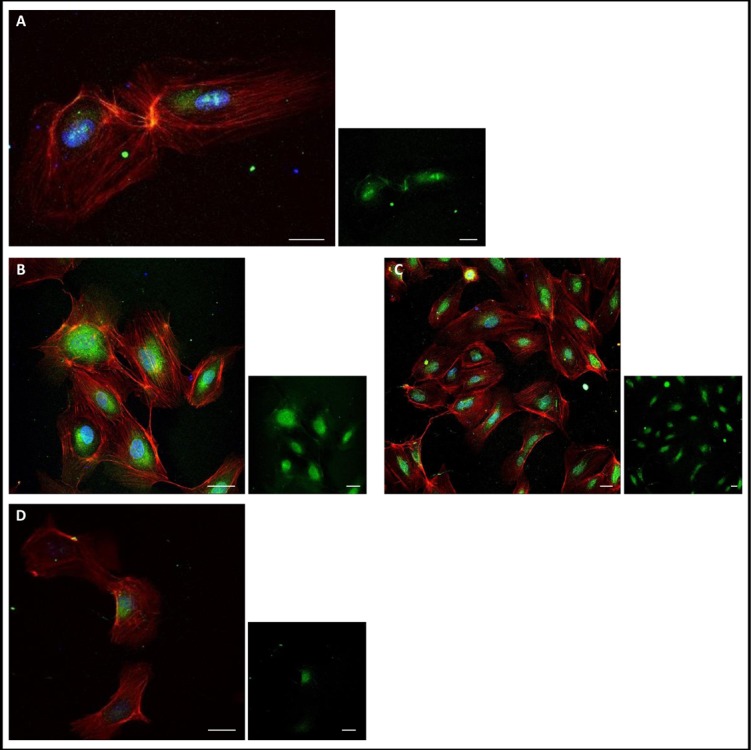
Peptide K108-K133 (26aa) is localized to the cell (A): 10 min of incubation with peptide K108-K133-peptide was located to the the cytoplasm surrounding the nucleus (green). (B): 2 h of incubation with peptide K108-K133-the peptide was accumulated in the cytoplasm surrounding the nucleus (green). (C): 8 h of incubation with peptide K108-K133-the peptide was accumulated in the cytoplasm surrounding the nucleus (green). (D): 24 h of incubation with peptide K108-K133-the peptide had minor presence inside the cell (green). Scale bar: 20μm

Peptide K108-K133 was labeled with green fluorescence. This procedure aallowed us to track the fate of the peptide during treatment of HUVE cells. We demonstrated that following 10 min of incubation with HUVE cells this peptide was visible in the cytoplasm surrounding the nucleus (Fig. 5). The peptide accumulated in the cytoplasm after 2 and 8 hours, but after 24h the cellular signal was less pronounced (Fig. 5).

#### 5.5.4 Peptide K108-K133 co-localize with angiogenin

**Figure 6:**
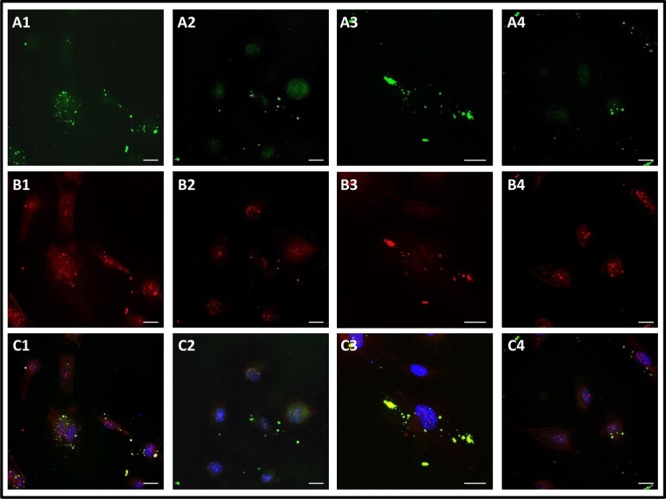
Peptide K108-K133 co-localize with angiogenin in HUVECs (A): peptide K108-K133 (green). (B): anti-angiogenin (red). (C): The integration of A and B (nucleus in blue, peptide in green and angiogenin in red). (A1): 10 min of incubation with the peptide and angiogenin– most of the peptide was localized outside the cell (green). (A2-A3): 2 h of incubation with the peptide and angiogenin– some of the peptide was observed in the cytoplasm surrounding the nucleus and some was located outside the cell (green). (A4): 8 h of incubation with the peptide and angiogenin– some of the peptide was observed in the cytoplasm surrounding the nucleus and some was located outside the cell (green). (B1): anti-angiogenin, 10 min of incubation with the peptide and angiogenin-angiogenin was located in the nucleus, the cytoplasm surrounding the nucleus, on the actin fibers and outside the cell (red). Same angiogenin localization was observed after 2 and 8 h of incubation with the peptide and angiogenin (B2, B3: 2 h. B4: 8 h) (red). (C1-C4): angiogenin and peptide K108-K133 generally co-localized mainly outside the cell after 10 min (C1), 2 h (C2, C3) and 8 h (C4).

In the presence of peptide K108-K133 and angiogenin we observed co-localization of both molecules (Fig. 6), similar to the co-localization observed with trT2-50 and angiogenin [25]. This finding suggests that both molecules bind (or compete) for similar cellular epitopes as previously reported for ACTIBIND [22]. Moreover, the addition of angiogenin to the cultured cells resulted in a reduction of the peptide signal inside the cell compared with the signal observed for the peptide alone (Fig. 6).

## DISCUSSION

We and others [3,10,22,23,25,28] have previously reported that the biological activity of T2-RNases is independent of their ribonuclease activity. However, we have shown that their actin binding capacity is essential. In line with our previous reports, we demonstrate herein that trT2-50m lacking the previously reported putative actin binding peptide [26,27] and the newly designed peptides based on hRNASET2 structure are able to bind actin and concomitantly inhibit angiogenesis. Actin binding ability was demonstrated using the immobilized actin solid phase assay and the BIAcore system. The antiangiogenic effect of selected actin-binding molecules was demonstrated by the significant inhibitory activity exerted on angiogenin- and VEGF-induced HUVE cell tube formation *in vitro* and in the *ex ovo* CAM assay. In addition, the immunofluorescent labeled peptide K108-K133 (26aa) rapidly underwent translocation towards the cell cytoplasm and nucleus of HUVECs. Angiogenin delayed the actin binding peptide penetration to the cell, suggesting that they may bind or compete for similar cellular epitopes.

Application of RNases toward diagnosis and treatment of diseases are suggested to be RNase-based mechanisms [29,30]. On the other hand, in members of the RNase T2 family, biological activity is not related to their ability to degrade RNA, as previously reported for ACTIBIND [3,22], for the full-length human RNASET2 [10] and for the truncated version (trT2-50) [25]. These results are consistent with those reported by Acquati et al (2005) for human RNASET2, in which a double point mutation at the catalytic site did not suppress its anti-cancer effect [23]. In this report, we clearly demonstrate that the RNase activity is not necessary for the actin binding and antiangiogenic activity of RNASET2.

A protein-protein docking study was recently conducted in order to inter-molecularly characterize the anticancerous property of *A. niger* RNase (ACTIBIND) [26]. The efficient binding of *A. niger* RNase and human actin revealed that the proteins could form hydrogen bond networks involving active amino acid residues, specifically, S E D A T Y K [26]. The ACTIBIND region that was suspected to be involved in the ability to bind actin was identified to T149-K170 [26]. Based on that, we identified the respective hRNASET2 homologous sequence, which is E120-V141. Regarding this region's structures of ACTIBIND and hRNASET2, we found high similarity at Q107-C160 and V136-C191, hRNASET2 and ACTIBIND numbering, respectively. These structures are composed of 3 helixes that we thought may play a central role in the ability to bind actin. Based on this we were able to suggest that the sequence suggested by Gundampati et al. 2012 [26] is not essential to bind actin. To prove this, we designed a truncated protein identical to trT2-50 but missing the 22 homologous amino acids (E120-V141). This truncated protein (named trT2-50m) was indeed found to bind actin similarly to trT2-50 and hRNASET2. Additionally trT2-50m displayed antiangiogenic activity as well. We therefore suggested that this region is not essential for the actin binding and antiangionenic activities of hRNASET2.

We further generated a peptide library consisting of 29 peptides based on the structural analysis and the three helixes suspected to play a role in actin binding. The design of the peptides reflected the helical wheel prediction analysis that was performed on all three helixes. All 29 peptides were tested for their ability to bind actin. For that purpose, we performed solid-phase actin binding assay (ELISA) and BIAcore analysis. These two assays demonstrated that trT2-50, trT2-50m, peptide A103-Q159 and peptide K108-K133 bound actin with similar affinities. All the other designed peptides did not have any significant binding activity towards actin. For the full length protein it was impossible to perform the ELISA assay probably due to steric disturbance, but in BIAcore analysis, the measured affinity was similar to the above molecules indicating that the three proteins (hRNASET2, trT2-50 and trT2-50m) and the two peptides (A103-Q159 and K108-K133) are similar in their ability to bind actin.

Peptide A103-Q159 comprises three helixes (Fig. 1B) whereas peptide K108-K133 consists of the first two helixes (Fig. 1B). This may indicate that the first two helixes are of fundamental importance for the actin binding, but, trT2-50m which is missing the second helix, (Fig. 1B) maintained its ability to bind actin. In addition, peptide E120-Q159 (40aa), consisting of helix 2 and 3, (Fig. 1B) is able to bind actin with lower affinity. We therefore suggest that the first helix is indeed of fundamental importance for the actin binding but that the second helix may be replaced with the third and still maintain the ability to bind actin. Peptide A103-V141 (39aa) consists of the first two helixes but has an additional loop at the C' terminal. This peptide binds actin with lower affinity than the two aforementioned peptides which may indicate that the loop at the C' terminal interrupts the interaction with actin.

We demonstrated that peptides A103-Q159 and K108-K133 have the ability to inhibit angiogenesis using HUVEC tube formation assay and using *ex ovo* CAM assay. We further demonstrated that the angiogenic-related inhibition is completely dependent on the peptide's ability to bind actin. We clearly showed that peptides able to bind actin were also able to inhibit tube formation, whereas peptides that failed to bind actin did not affect tube formation.

Cancer is a tissue-based disease; therefore, the integrity of tissue architecture is a major constraint toward cancer growth [19]. Surface actin in endothelial cells has been demonstrated to serve as a receptor for angiogenin, plasminogen and tissue plasminogen activator, proteins which play central role in angiogenesis and tumor progression [31-33]. We suggest that hRNASET2 or its actin-binding peptide derivatives may compete with each of these proteins that bind endothelial cell surface actin therefore inhibiting cell invasiveness and migration. This is supported by our previous study that established a competitive mechanism of ACTIBIND and angiogenein on actin binding [22].

Our previous immunofluorescence experiments demonstrated that RNASET2 endogenous protein is expressed in both the nuclei and the cytoplasm of HUVECs and readily leaves these cell compartments following exposure to trT2-50. We surmise that the aim of this translocation is to reach actin membrane epitopes [25]. A similar response was demonstrated for stress-induced melanocytes and keratinocytes that appeared to stimulate a dynamic movement of RNASET2 from the perinuclear region to the cellular membrane and out to the extracellular space [34].

We additionally demonstrate herein (see Figure 5) that in HUVE cells following 10 min incubation with peptide K108-K133, this molecule readily enters the cytoplasm. The peptide is then accumulated in the cytoplasm surrounding the nucleus after 2 and 8 hours, but after 24 hours the cellular signal was less pronounced. We therefore suggest that this peptide quickly diffuses into the cell and accumulates in the cell, but after 24 hours probably translocates to bind membrane-associated actin. In addition, peptide K108-K133 and angiogenin were co-localized, similar to the co-localization observed with trT2-50 and angiogenin [25]. In the presence of angiogenin the peptide signal inside the cell was less pronounced than the signal observed with the peptide alone. Angiogenin probably inhibited the entrance of the peptide to the cell.

**In conclusion**, the present findings demonstrate novel actin-binding peptides derived from the hRNASET2 protein. We demonstrate that the potency of these peptides to inhibit angiogenesis directly depends on their ability to bind actin. We suggest that these newly designed peptides may serve as potential antiangiogenic therapeutic agents.

## MATERIALS AND METHODS

### trT2-50m cloning

The *human trT2-50m* synthetic gene was introduced into a pUC57 vector (Genscript Corporation, Piscataway, NJ, USA). The pUC57 containing the gene and the pHIS-Parallel3 vector were cleaved with *Nde*I and *Bam*HI restriction enzymes (Fermentas AB, Vilnius, Lithuania) in order to allow cloning. The DNA fragments after restriction were isolated on 1% agarose gel and extracted using the Wizard SV Gel and PCRA Clean Up System (Promega, Madison, WI). The isolated DNA fragments were ligated into the cleaved plasmids. The ligation was carried out at 16°C overnight in rapid ligation buffer (Bio-Lab LTD). Ligase (Fermentas AB) was inactivated at 65°C for 10 min.

### Transformation into *E. coli*

Competent *E. coli* cells were prepared as described in [35] and maintained at −70°C. The ligation product (8 μl) was transformed into competent *E. coli* DH5α by heat shock. Bacteria were then seeded on LB ampicillin containing plates. For plasmid extraction, a single colony was selected and inoculated in 5 ml LB medium, at 37°C, in a rotary shaker (250 rpm). Plasmid DNA extraction was carried out with the Jetquick Plasmid Miniprep Spin Kit (Genomed Inc., Gmgh Lohne, Germany) and used to transform 60 μl *E. coli* BL21 (DE3) competent cells using heat shock. Bacteria were then seeded on LB ampicillin contacting plates. Plasmids were sequenced at the Center for Genomics Technologies, The Institute of Life Science, The Hebrew University of Jerusalem, Israel.

### Truncated Protein expression

*E. coli* BL21(DE3) colonies containing the trT2-50m gene in the pHIS-Parallel3 vector were cultured overnight (37°C and 250 rpm) in 10 ml LB medium. Culture samples (6 ml) were then transferred to 400 ml LB medium and grown to OD_600_ =0.6-0.8. Protein expression was induced by addition of 1 mM isopropyl β-D-thiogalactopyranoside (IPTG) (Dushefa, Haarlem, The Netherlands) for 3 h at 37°C, 250 rpm. The cells were then harvested by centrifugation at 14,000 g for 10 min at room temperature.

### Cell lysis

After centrifugation, bacterial cell pellets of 100 ml cultures were resuspended in 25 ml lysis buffer containing 20 mM phosphate buffer, 8 M urea, 0.1 NaCl, 1 mM EDTA (pH 8) and 2 mg/ml complete protease inhibitor (Roche Diagnostics, Mannheim, Germany). The harvested cells were then stirred for 2 h at 4°C. The lysates were centrifuged at 14,000 g for 30 min at room temperature and the supernatant was filtered (Whatman® FP30/0.2 μm, cellulose acetate filter).

### Protein purification

Filtrated bacterial lysate containing the recombinant protein was loaded, in lysis buffer, onto a 1 ml HisTrap Ni-Sepharose affinity column (GE-Healthcare Bio Sciences AB, Uppsala, Sweden). Proteins were eluted with an imidazol gradient (5-500 mM), prepared in equilibration buffer containing 20 mM sodium phosphate (pH 8.0), 1 M NaCl, 8 M urea and 5 mM β-mercaptoethanol, at a flow rate of 1 ml/min using GE-Healthcare's ACTAprime plus FPLC system (GE-Healthcare Bio Sciences AB). The fractions collected from the peak (~ 75 mM imidazol ) were analyzed by 12.5% SDS-PAGE followed by Coomassie R250 staining. Protein containing fractions were pooled for refolding.

### Protein refolding

The purified protein was refolded by dialysis against 20 mM Tris solution (pH 12.0). Dialysis solution was exchanged once per hour for four hours and then left overnight at room temperature. The same procedure was then used with 20 mM Tris solution (pH 10) and finally with 20 mM ammonium bicarbonate buffer (pH 8). The refolded protein was lyophilized and kept at 4°C.

### Peptide library

Based on Gundampati et al. 2012, and Kumar et al. 2013 [26,27] findings who claimed that they have identified the actin-binding sequence of ACTIBIND we subsequently searched for an homologous sequence in the human RNASET2 sequence and identified it. We then generated a peptide library containing 29 peptides based on this sequence and based on structural analysis. Synthetic peptides were ordered from Genemed Synthesis Inc. (Tx USA, by HyLabs, Israel) or from GenScript Corporation (Piscataway, NJ). Additionally, we tested the ability of each of the synthetic peptides to bind actin and to inhibit angiogenesis.

### Actin binding solid phase assay

96-well plates (MaxiSorp® flat-bottom 96-well plate, Fisher Scientific Inc., Fair Lawn, NJ) were coated with 500 ng actin in 100 μl carbonate-bicarbonate buffer (pH 9.5) for 1 h at 37°C. The plate was washed once with TBS and then blocked with 3% BSA in 200 μl TBS buffer at 37°C, for 1 h. Wells were then washed once with 250 μl TBS. trT2-50, trT2-50m or each of the 29 peptides were added at 1:2 dilutions in 100 μl TBS, starting from 500 ng/well, incubated for 1 h at 37°C and then washed three times with TBS containing 0.1% Tween-20 (TBST). Each well was then treated with 100 μl rabbit anti-trT2-50 diluted 1:500 in TBS and incubated for 1 h at 37°C. The wells were washed three times with TBST and then incubated with 100 μl goat anti-rabbit IgG-HRP (Jackson ImmunoResearch, West Grove, PA) diluted 1:10,000 in TBS, for 1 h at 37°C. Wells were then washed twice with TBST, and once with TBS before 100 μl substrate (1-step Ultra TMB-ELISA, Pierce, Fisher Scientific Inc.) were added. Absorbance at 655 nm was measured 10 min thereafter using a Power Wave 200 Microplate Scanning Spectrophotometer (Bio-Tek Instrument, Winooski, VT). Affinity was evaluated by double reciprocal plot [36,37].

### Surface Plasmon Resonance (SPR) of actin binding

SPR was performed on a BIAcore 3000 instrument (BIAcore, Uppsala, Sweden). Actin was diluted in 100 mM CH3COONa pH 4.6 to a final concentration of 20μg/ml (200μl total) resulting in ~1ng immobilized actin on a CM5 BIAcore sensor chip (GE Healthcare Bio-Sciences AB) using the standard BIAcore amine coupling chemistry protocol [38]. The CM5 chip allows four separate flow cells to be operated in the BIAcore 3000 instrument used in these experiments. Coupling is achieved by activating the surface of the sensor chips with equal volumes of 50 mM *N*-hydroxysuccimide (NHS)/200 mM *N*ethyl-*N*-(3-diethylaminopropyl)-carboiimide (EDC) (BIAcore, AB, Uppsala, Sweden) to form activated carboxyl groups. In the BIAcore protocol the binding is believed to be between carboxyl groups on the surface and amine groups on the protein. The actin solution (15 μg/ml) was then run over the chip for 5 min at a rate of 10 μl/min, where the amine groups on protein react with the activated esters. In the final stage of immobilization, the surface was blocked by 1M ethanol amine, pH 8.0. The binding assay was performed by injecting the hRNASET2, trT2-50, trT2-50m or peptides solutions in running buffer (HBS-10mM Hepes, 150mM Sodium Chloride, 3mM EDTA, 0.005% Polysorbate 20) at 5 different concentrations at a flow rate of 10 μl/min at 25°C. Injections were performed simultaneously over all four channels and blank surface a plain dextran matrix (channel 1) was used as control. The net signal was obtained by subtracting the blank signal from the signal of the immobilized surface. The association phase for protein or peptides binding to actin was followed for 4 min, and dissociation phases were monitored for 3 min. Surface regeneration between consecutive binding cycles included a 1 min injection of 1 mM NaOH. The response was monitored as a function of time (sensorgram) at 25°C. Multi-concentration data were globally fit using BIA evaluation 3.2 software.

The strength of a two molecule interaction is characterized by the equilibrium dissociation (binding) constant KD =[P][L]/[PL], where [P] is the concentration of free protein (or peptide), [L] the concentration of ligand and [PL] the concentration of the complex. At equilibrium, K_D_ is related to the rate of complex formation (described by the association rate constant, k_a_) and the rate of breakdown (described by the dissociation rate constant, k_d_ ), such that K_D_ =k_a_/k_d_. A high affinity interaction is characterized by a low K_D_, rapid recognition and binding by high k_a_, and stability of complex formation by low k_d_.

### Angiogenesis assays

### HUVEC tube formation assay

Freshly isolated HUVEC were maintained in endothelial cell growth medium (EGM) supplemented with SingleQuots (EGM BulletKit CC-3124, Lonza). They were then plated in a 96-well plates (14 × 10^3^ cells/well) previously coated with growth factor-depleted Matrigel (Becton-Dickinson, Bedford, MA) in M199 medium supplemented with 20% FBS, 1% glutamine, 1% antibiotic–antimycotic solution (Biological Industries) and 50 U/100 ml heparin (Biomedical Technologies Inc., Stoughton, MA). Cells were treated with trT2-50m (2 μM), peptides or PBS, in addition to angiogenin (R&D Systems, Inc. Minneapolis, MN), or VEGF (Protein Laboratories, Rehovot, Israel) (1 μg/ml each). After 8 h of incubation at 37°C, the plates were photographed and the extent of tube formation was counted using Image J (NIH, Bethesda, MD) software. Five individual determinations were performed for each treatment. Statistical analysis was performed using one way ANOVA for multiple comparison and 2-sample t-test.

### *Ex ovo* CAM angiogenesis assay

Fertilized chicken eggs were incubated horizontally at 37°C and humidity of 60 - 62% and cracked into Petri dishes at embryonic day 4. Incubation was continued under the same conditions. On day 8, sterile filter paper disks (5.5 mm in diameter) were layered on top of the CAM and soaked either with 5 μl PBS or 3 μg hRNASET2, trT2-50, peptide A103-Q159 or peptide K108-K133. Treatment was given every day for four days, then, the number of blood vessels around the treated disks was counted. (For hRNASET2 and trT2-50, N=3; for peptide A103-Q159, peptide K108-K133 and PBS, N=5).

### Immunofluorescence

Cells were cultured on PBS-covered slides in 12-well plates with 0.1% pork gelatin (Sigma-Aldrich) and were incubated with peptide K108-K133 or angiogenin. Cells were fixed with 3% Paraformaldehyde (PFA) (Merck Millipore, Dermstadt, Germany) containing 0.5% triton, washed three times with PBS and blocked with 5% donkey serum (Jackson ImmunoResearch). Rabbit anti-angiogenin (Merck Millipore) was added (1:100 dilution; prepared in 5% donkey serum) and incubated overnight at 4°C. After washing three times with TBST, the slides were incubated for 1 h with Alexa 488-conjugated anti-rabbit antibody (Invitrogen Life Technologies) and phalloidin tetramethylrhodamine B isothiocyanate–conjugated anti-rabbit antibody (Sigma-Aldrich), and then washed, and mounted with a mixture containing 30% mounting medium, 4′,6-diamidino-2-phenylindole (DAPI) (Santa Cruz Biotechnology Inc., Santa Cruz, CA) and 70% fluoromount (Sigma-Aldrich). The slides were viewed under a Leicactr4000 laser scanning confocal microscope.

## SUPPLEMENTARY TABLE AND FIGURE



## Abbreviations

HUVEC: human umbilical vein endothelial cell
RNASE: ribonuclease
IPTG: isopropyl β-Dthiogalactopyranoside
EDTA: ethylenediaminetetraacetic acid
SAB: sample application buffer
TBS: tris buffer saline
TBST: tris buffer saline with tween-20
SDSPAGE: sodium dodecyl sulfate polyacrylamide gel electrophoresis
FBS: fetal bovine serum
PBS: phosphate buffered saline
