# Supplementary material for: Human RNASET2 derivatives as potential anti-angiogenic agents: actin binding sequence identification and characterization
Source: Oncoscience. 2014 Nov 26;2(1):31–43. doi: 10.18632/oncoscience.100 (PMC4341462; doi:10.18632/oncoscience.100)
Supplement: Supplementary file 1 [file oncoscience-02-0031-s001.pdf]

Name	Sequence
hRNASET2	**DKRLRDNHEWKKLIMVQHWPETVCEKIQ NDCRDPPDYWTIHGLWPDKSEGCNRSWPF NLEEIKDLLPEMRAYWPDVIHSFPNRSRFW KHEWEKHGTCAAQVDALNSQKKYFGRSL ELYRELDLNSVLLKLGIKPSINYYQVADFK DALARVYGVIPKIQCLPPSQDEEVQTIGQIE LCLTKQDQQLQNCTEPGEQPSPKQEVWLA NGAAESRGLRVCEDGPVFYPPPKKTKH**
Peptide E120-V141 (22aa)	**ELDLNSVLLKLGIKPSINYYQV**
Peptide S135-V141 (7aa)	**SINYYQV**
Peptide E120-Q159 (40aa)	**ELDLNSVLLKLGIKPSINYYQVADFKDALA RVYGVIPKIQ**
Peptide A103- Q159 (57aa)	**ALNSQKKYFGRSLELYRELDLNSVLLKLGI KPSINYYQVADFKDALARVYGVIPKIQ**
Peptide D75- D102 (28aa)	**DVIHSFPNRSRFWKHEWEKFGTCAAQVD**
Peptide A103- R119 (17aa)	**ALNSQKKYFGRSLELYR**
Peptide K108- R119 (12aa)	**KKYFGRSLELYR**

Peptide A103- V141	**ALNSQKKYFGRSLELYRELDLNSVLLKLGI KPSINYYQV**
Peptide K108- E120 (13aa)	**KKYFGRSLELYRE**
Peptide K108- L121 (14aa)	**KKYFGRSLELYREL**
Peptide K108- D122 (15aa)	**KKYFGRSLELYRELD**
Peptide K108- L123 (16aa)	**KKYFGRSLELYRELDL**
Peptide K108- N124 (17aa)	**KKYFGRSLELYRELDLN**
Peptide K108- S125 (18aa)	**KKYFGRSLELYRELDLNS**
Peptide K108- V126 (19aa)	**KKYFGRSLELYRELDLNSV**
Peptide K108- L127 (20aa)	**KKYFGRSLELYRELDLNSVL**
Peptide K108- L128 (21aa)	**KKYFGRSLELYRELDLNSVLL**
Peptide K108- K129 (22aa)	**KKYFGRSLELYRELDLNSVLLK**
Peptide	**KKYFGRSLELYRELDLNSVLLKL**

K108- L130 (23aa)	
Peptide K108- E120+ N124 -K133 (23aa)	**KKYFGRSLELYRE___NSVLLKLGIK**
Peptide K108- K133 (26aa)	**KKYFGRSLELYRELDLNSVLLKLGIK**
Peptide G112- K133 (22aa)	**GRSLELYRELDLNSVLLKLGIK**
Peptide R113- K133 (21aa)	**RSLELYRELDLNSVLLKLGIK**
Peptide S114- K133 (20aa)	**SLELYRELDLNSVLLKLGIK**
Peptide K108- I132 (25aa)	**KKYFGRSLELYRELDLNSVLLKLGI**
Peptide K108- G131 (24aa)	**KKYFGRSLELYRELDLNSVLLKLG**
Peptide K109- K133 (25aa)	**KYFGRSLELYRELDLNSVLLKLGIK**
Peptide Y110- K133 (24aa)	**YFGRSLELYRELDLNSVLLKLGIK**
Peptide F111- K133	**FGRSLELYRELDLNSVLLKLGIK**

(23aa)	
trT2-50 truncated protein E49-Stop codon	**EGCNRSWPFNLEEIKDLLPEMRAYWPDVIH SFPNRSRFWKHEWEKHGTCAAQVDALNSQ KKYFGRSLELYRELDLNSVLLKLGIKPSINY YQVADFKDALARVYGVIPKIQCLPPSQDEE VQTIGQIELCLTKQDQQLQNCTEPGEQPSP KQEVWLANGAAESRGLRVCEDGPVFYPPP KKTKH**
trT2-49m (truncated protein, missing Peptide E120-V141 sequence)	**EGCNRSWPFNLEEIKDLLPEMRAYWPDVIH SFPNRSRFWKHEWEKHGTCAAQVDALNSQ KKYFGRSLELYR______________________AD FKDALARVYGVIPKIQCLPPSQDEEVQTIGQ IELCLTKQDQQLQNCTEPGEQPSPKQEVW LANGAAESRGLRVCEDGPVFYPPPKKTKH**

Supplementary Table 1: Peptides library- sequences of all 29 synthetic peptides and the three proteins (hRNASET2, trT2-50 and trT2-50m).

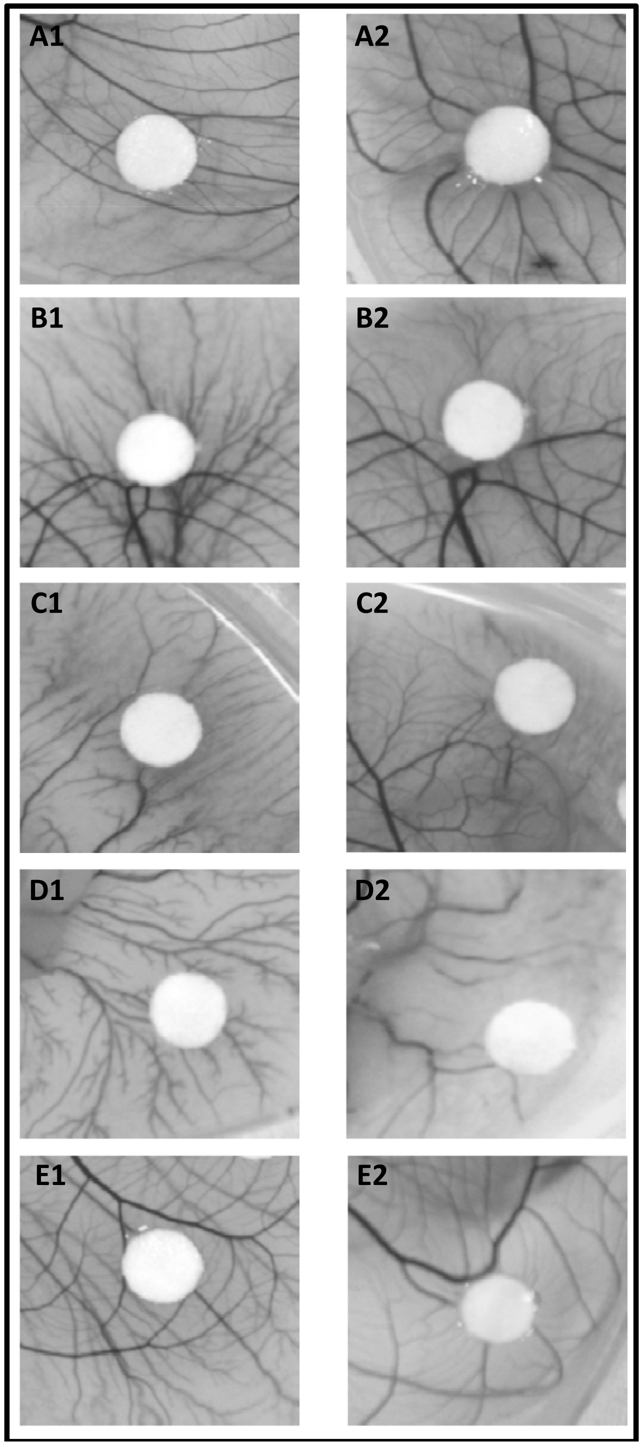

Supplementary fig. S1

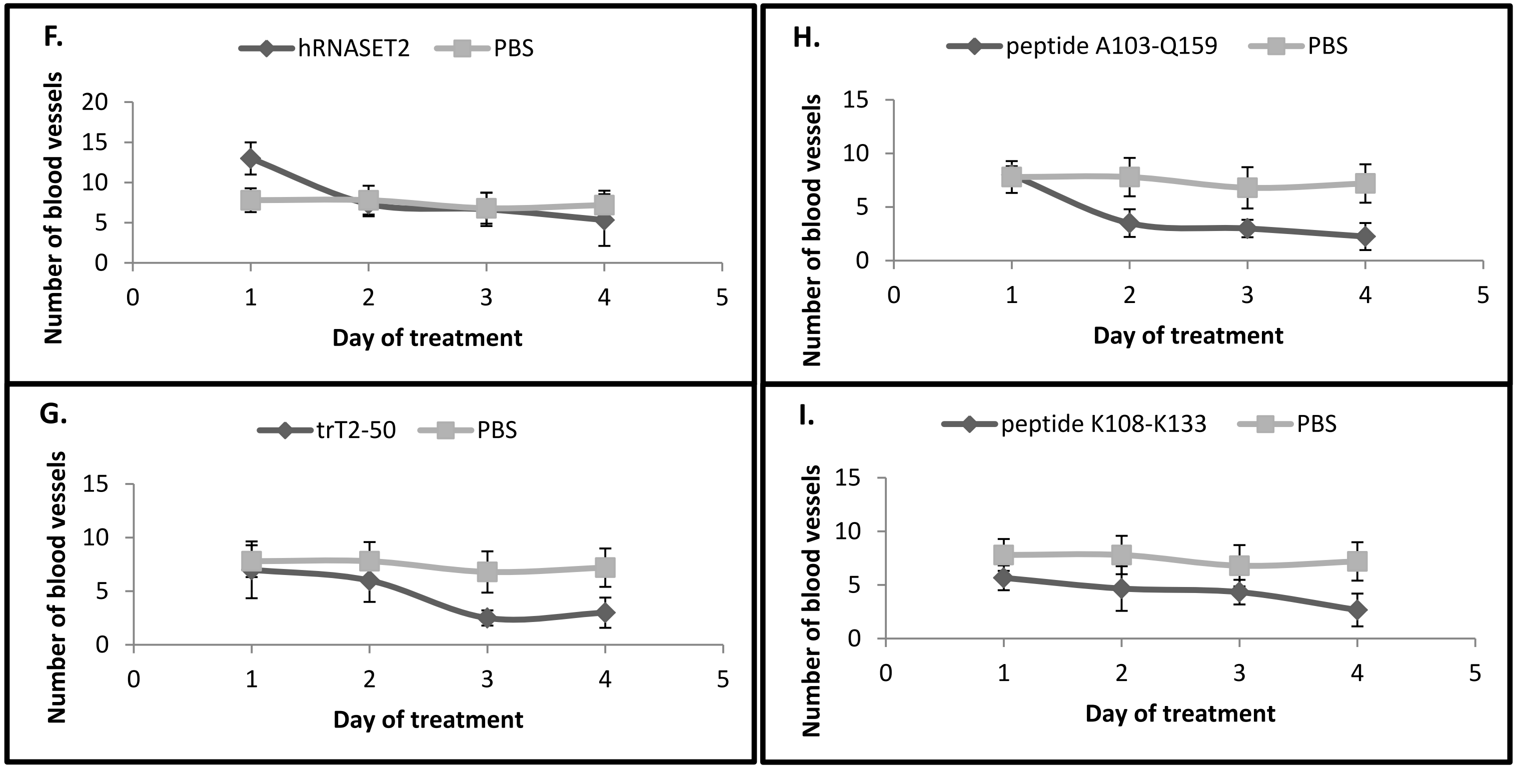

Supplementary fig. S1
